# IN-SILICO dynamic analysis of Sulawesi propolis as anti-endometriosis drug: Interaction study with TNF alpha receptor, NF-kB, estrogen receptor, progesterone receptor and prostaglandin receptor

**DOI:** 10.1016/j.amsu.2021.102459

**Published:** 2021-06-17

**Authors:** Herbert Situmorang, Andon Hestiantoro, Sigit Purbadi, Darin Flamandita, Muhamad Sahlan

**Affiliations:** a Department of Obstetrics and Gynecology Faculty of Medicine Universitas Indonesia – Dr Cipto Mangunkusumo National Referral Hospital, Jl. Salemba Raya No. 6, Central Jakarta, Jakarta Capital Special Region, 10430, Indonesia; b Department of Chemical Engineering, Faculty of Engineering, Universitas Indonesia, Jl. Fuad Hasan, Pancoran MAS, Kukusan, Beji, Depok City, West Java, 16424, Indonesia

**Keywords:** Docking, In silico, Propolis, Endometriosis, TNF alpha Receptor, NF-kB, Estrogen receptor, Progesterone receptor, Prostaglandin receptor

## Abstract

**Introduction:**

Endometriosis is a disease that impacts around 10% of all women in reproductive age, with pelvic pain and infertility as its main clinical features. Current medical treatment targeting lowering estrogen activity has not shown sufficient result due its side effects and reproductive function suppression. Propolis has been widely studied, showing anti inflammation and pro-apoptosis property, that could potentially be used in the treatment of endometriosis. This study investigates the interaction between Sulawesi Propolis’ active components and receptors and protein related to endometriosis pathogenesis.

**Methods:**

Active components of Sulawesi Propolis were initially identified with their targeted protein receptors. Lipinski rules were used to screen potential components. The ligands and proteins were tested using Autodock program to predict the most active compound and possible binding sites between propolis and some target proteins associated with inflammatory and apoptotic activity in endometriosis models. Receptor modelling is then performed using Swiss-Model.

**Results:**

These active components of Sulawesi Propolis showed a strong binding potential towards TNF- α, NF-kb, Estrogen-α, Estrogen-β, progesterone B, PGE2 EP2 and EP3 subtype respectively: Sanggenon C, Sanggenon H, Epicryptoacetalide, Chrysin-7-O-β-D-glucopyranodside, Irilone, Polydatin and Epicryptoacetalide. Compared to its negative ligand, Sulawesi Propolis displayed a stronger binding capacity to TNF-α, Estrogen-α, and Progesterone B receptors.

**Conclusion:**

Sulawesi Propolis has the ability to interact with receptors related to reproductive function, apoptotic reactions and inflammatory processes, a significant factor associated with the pathogenesis of endometriosis.

## Highlights

• Endometriosis remains a significant health problem recently, and affects about 10% of all women in reproductive age.
• Current medical treatments targeting on lowering estrogen activity have drawbacks due to their side effects and reproductive function suppression during treatments.
• Propolis has been widely studied, showing its biological properties that could potentially be used as a therapeutic modality in endometriosis.
• Sulawesi Propolis has the ability to interact with receptors related to reproductive function, apoptotic activity and inflammatory processes, significant factors associated with the pathogenesis of endometriosis.

## 1 Introduction

Endometriosis found in women of childbearing age is still a big problem nowadays. With an incidence of around 10% of all female ages, complaints about chronic pelvic pain and infertility can cause physical, mental and social problems for patients. However, the current medical treatment showed unsatisfactory result in overcoming problems, even aggravating the patient's suffering due to failure to meet patient expectations.

The inflammatory reaction to endometrial tissue outside the uterine cavity causes various problems, especially pelvic pain and subfertility. Nuclear factor kappa-light-chain-enhancer activated B cells (NF-kB) pathway increases the expression of inflammatory mediators such as interleukin (IL) -1B, IL-6, IL-8 and Tumor Necrotizing Factor (TNF) alpha. The release of these inflammatory mediators accelerates the prolonged inflammatory process and is responsible for the reduced activity of endometriosis cell apoptosis [1].

Medical treatment targeting single pathogenesis mechanism only, does not bring satisfactory results. Surgery alone cannot seem to solve endometriosis without a long-term postoperative treatment strategy to prevent the recurrence of this disease [2]. Besides, endometriosis medical treatment is still based on hormonal drugs that suppress the estrogen's action, as the primary pathway for its various pathogenesis. This approach results in attenuation of folliculogenesis and ovulation, which negate the possibility of being conceived during the treatment [3]. Therefore, there is a high urgency in providing endometriosis medication that can be used long-term with minimum side effect, and does not alter the hypothalamus-pituitary-ovarian pathway.

One of the natural ingredients that has been widely studied for its health benefits is Propolis. Propolis from *Tetragonula aff. biroi* originating from Luwu District, South Sulawesi has been investigated to have anti-oxidant and anti-inflammatory effects as well as to have a pro-apoptotic effect on various cancer line cells [4]. The effect of the Propolis active substances, which target various pathogenesis pathways for endometriosis, has the potential to be used as medical therapy for endometriosis. However, there is no data on the use of Propolis in endometriosis. This signifies the importance of research that can prove the benefits of Propolis Indonesia as a medical treatment in endometriosis that increases apoptotic activity, decreases the inflammatory activity, and does not interfere with ovulation.

The process of developing drugs from natural ingredients is known be lengthy (11–16 years) [5]. Thus, to accelerate the process of drug discovery, a variety of computer software has been used called the in-silico research model. In investigating its efficacy, an in-silico study was conducted to determine Propolis active components that can interact with endometriosis-related receptors such as TNF-alpha, NF-κB, estrogen, progesterone, and prostaglandin receptors.

## 2 Material and methods

The previous study has elucidated the active components of Indonesian Propolis. These compounds are 2-Methoxykurarinone, Kurarinone, Chrysin-7-O-β-d-glucopyranoside, Flavenochromane B, Ginkgol, Irilone, Leachianone A, Icaritin, Sanggenon C, Scutellarein, Cimicifugic acid, Demethoxycurcumin, Dendrocandin B, Ricinoleic acid, Polydatin, Epicryptoacetalide, Caesalpins J, Ginkgetin, Lupinifolin, Rhamnetin, Sanggenon H, Shogaol, 4- Hydroxy ginkgolic acid, 9,16-Dioxyhydroxy-10,12,14-triene-18 carbonic acid, and 4′-O-Methylbrazilin [6].

Twenty-five compounds in Propolis were tested for screening, predicting which compounds might be developed into drugs using Lipinski's rule of five. Three-dimensional shapes of compounds were made and stored as a ligand with PBD (Protein Data Bank) extension. Protein or receptor model involved in endometriosis pathogenesis, i.e. TNF alpha receptor, NFĸB receptor protein, estrogen receptor alpha, estrogen receptor beta, prostaglandin E2 receptor subtype EP2 and EP3 were to be tested. A receptor modelling using Swiss Model from Uniprot sequencing was created for receptors whose model is unavailable in PDB. These ligands and proteins were tested using the standard Lamarckian algorithm in the Autodock program. The binding energies (ΔG), inhibitor concentration (Ki), hydrogen bond (H-bond) involved in the ligand-receptor complex formation were determined.

## 3 Results

Following Lipinski's rules of five, 22 out of 25 active compounds from *Tetragonula aff. biroi* bee colony propolis showed a strong binding potential with endometriosis-related receptors such as TNF-⍺, NFĸB, estrogen α, estrogen β, progesterone A, progesterone B, and prostaglandin E2 as shown in Table 1.

**Table 1:** Docking results (ΔG) of propolis active compounds affinity to receptors associated endometriosis pathogenesis.

No	Active Compounds	TNF Alpha	NF-kB	Estrogen Alpha	Estrogen Beta	Proges-terone B	PGE2 EP3 Subtype	PGE2 EP2 Subtype
1	2-methoxykurarinone	−7,9	−6,5	−6,0	−5,1	−4,9	−8,5	−5,6
2	Kurarinone	−8,-	−6,9	−5,7	−6,8	−5,1	−9,0	−6,0
3	Chrysin-7-O-β-d-glucopyranoside	−8,8	−8,7	−7,7	−9,1	−6,6	−10,3	−7,2
4	Flavenochromane B	−9,5	−8,4	−8,3	−7,5	−5,3	−5,9	−7,3
5	Ginkgol	−5,8	−5,8	−7,7	−7,4	−7,5	−7,4	−6,3
6	Irilone	−8,2	−8,6	−8,3	−7,3	−10,0	−9,3	−7,3
7	Leachianone A	−8,6	−7,1	−6,7	−6,1	−8,1	−9,3	−6,1
8	Icaritin	−7,8	−7,5	−8,1	−6,1	−7,2	−8,2	−5,6
9	Scutellarein	−6,8	−8,1	−8,2	−7,3	−8,6	−8,4	−6,2
10	Cimicifugic acid	−7,9	−7,9	−6,6	−7,5	−8,2	−9,2	−6,5
11	Demethoxycurcumin	−7,4	−7,9	−8,0	−7,4	−7,7	−10,3	−6,3
12	Dendrocandin B	−8,1	−7,5	−6,9	−6,6	−6,3	−7,2	−6,5
13	Ricinoleic acid	−5,6	−5,4	−7,1	−6,9	−6,8	−6,9	−5,4
14	Polydatin	−7,7	−7,6	−6,1	−6,8	−6,2	−10,7	−6,1
15	Sanggenon H	−8,8	−9,8	−8,3	−6,9	−8,3	−9,2	−7,3
16	Sanggenon C	−10,2	−8,7	+5,6	−4,9	−6,5	−5,4	−7,2
17	Epicryptoacetalide	−8,2	−8,5	−8,9	−7,7	−7,9	−9,7	−7,8
18	Shogaol	−5,8	−5,6	−7,0	−6,9	−6,9	−6,5	−5,5
19	Caesalpins J	−7,8	−6,8	−8,2	−7,0	−7,0	−6,6	−6,3
20	Ginkgetin	−9,9	−8,4	−6,5	−7,4	−6,0	−7,0	−7,0
21	Lupinifolin	−8,8	−8,3	−0,8	−6,3	−6,7	−9,0	−6,4
22	Rhamnetin	−7,7	−8,7	−8,1	−6,9	−8,4	−9,1	−6,8
	Native ligand	−8.23		−9,8	−7.9	−7.9		−7.96

All active components of Indonesia propolis showed a high binding potential (less than 0) to TNF-alpha receptors. This confirms other studies reported a good anti-inflammatory activity of Indonesia propolis [7]. Sanggenon C has the highest affinity with ΔG 10.2 KCal/mol, and Ki 0.03 μM. Meanwhile, in the NF-kB, Sanggenon H has the highest affinity with ΔG 9,8 KCal/mol, and Ki 0.07 μM^8^.

All active components of Indonesia Propolis showed a good negative-docking score to ER-α, except Sanggenon C, which showed a positive docking score. Epicryptoacetalide revealed the highest affinity to ER-α with ΔG 8,9 KCal/mol, and Ki 0.3 μM. Moreover, the highest docking score affinity to ER-β with ΔG 9,1 KCal/mol, and Ki 0.21 μM was shown by Chrysin-7-OBD-glucopyranoside [8].

Besides having the highest affinity to TNF-alpha receptor, Sanggenon C also signified the highest affinity to PR-B with ΔG 10 KCal/mol, and Ki 0.05 μM. Our study in PGE2 receptor (EP3 subtype), elucidated that Polydatin exhibited the highest affinity with ΔG 10,7 KCal/mol, and Ki 0.01 μM, and finally, Epicryptoacetalide displayed the highest affinity with ΔG 10,7 KCal/mol, and Ki 0.01 μM to PGE2 receptor (EP2 subtype), see Table 1.

Docking interactions were grouped to determine the Gibbs energy (ΔG) since the lower ΔG showed the conformational energy for the best docking value. Calculation of inhibitor concentration (Ki) was reported to determine the binding energy produced from docking as shown in Table 2. Each compound revealed a different conformation which was correlated with the binding energy value. After determining the active compound propolis that might show the highest affinity with the receptor related to the pathogenesis of endometriosis, then we made a receptor modeling in which the propolis compound might be able to bind to the receptor's active site.

**Table 2:** Summary of docking results, 3D visualization and hydrogen binding site.

Protein Target	Substance with Strongest Binding Affinity	ΔG in Kcal/Mol (Ki in μM)	H Bound	3D Visualization
TNF-alpha	Sanggenon C	−10,2 (0,03)	5 (Tyr151(A), Tyr119(B), Ser60(B), Ser60(B), Leu120(B))	
NF-kB p50/p65	Sanggenon H	−9,8 (0,07)	2 (Arg246(A), da18(D))	
ER-A	Epicryptoacetalide	−8,9 (0,30)	–	
ER-B	Chrysin-7-O-β-d-glucopyranoside	−9,1 (0,21)	4 (Asn234(A), Glu260(A), Val293(A), Tyr352(A))	
Progesterone Receptor B	Irilone	−10 (0,05)	1 (Tyr151(B))	
PGE2 EP3	Polydatin	−10,7 (0,01)	8 (Thr206(A), Tyr114(A), Ser336(A), Ser336(A), Gln339(A), Gln339(A), Gly102(A), Thr136(A))	
PGE2 EP2	Epicryptoacetalide	−7,8 (1.92)	–	

We utilized the protein complex structure from the protein data bank in which a compound binds in its receptor active site. The protein complex exhibited a great box with a center coordinate XYZ and tethering size area where the compounds bind to the receptor. Afterward, we replaced that compound with the propolis active compound and then put the propolis active compound to the active binding site as seen in Table 2. Therefore, when the propolis active compound was bound to the receptor-associated endometriosis pathogenesis, it was expected can inhibit the signaling pathway in endometriosis pathogenesis.

## 4 Discussions

Multiple pathways are involved in endometriosis pathogenesis, in which chronic inflammation is one of its significant manifestations. The endometriosis-associated inflammatory responses are dependent on increased activated macrophages and their secreted cytokines in peritoneal fluid. A local inflammatory microenvironment sustains endometriosis’ growth and maintenance through endometrial-peritoneal adhesion, invasion, angiogenesis, and proliferation. The inflammatory process in endometriosis further causes pelvic pain and infertility, two prominent symptoms of endometriosis.

The use of Propolis in endometriosis treatment has not been studied extensively. This study elucidates the molecular binding of propolis components to several targeted proteins known to be essential pathways of endometriosis. Sulawesi Propolis’ component has been analyzed in previous studies^4^**.** Molecular docking results revealed that the active components of Sulawesi Propolis have high binding potential to proteins involved in endometriosis pathophysiology, namely: TNF-alpha receptors, NF-kB p50/p65, estrogen receptors -alpha (ER-α) and -beta (ER-β), progesterone receptor B (PR-B) as well as prostaglandin receptors E2 with EP2 subtype and EP3 subtype.

Nuclear factor kappa-light-chain-enhancer of activated B cells (NFκB) is known to be one of the most important transcription factors that facilitate survival and growth of endometriosis cells in addition to the ERK1/2 pathway, and AKT. This NFκB pathway increases the expression of inflammatory mediators such as interleukin (IL)-1B, IL-6, IL-8, Regulated on Activation Normal T cell Express and Secreted (RANTES), Intercellular adhesion molecule (ICAM) 1, Monocyte Chemoattractant Protein (MCP) 1, Cyclooxygenase (COX) 2, Macrophage migration inhibition (MIF), Matrix Metallo Protein (MMP) 9, and Tumor Necrosing Factor (TNF). These will trigger a prolonged inflammatory process in endometriosis cells and surrounding tissue. Besides, this NFκB pathway is also responsible for the reduced activity of endometriosis cell apoptosis.

Our study shows that Sanggenon H has the highest docking score to NF-kB. Sanggenon H has been found in the root of *Morus. Morus alba* L. and *M. nigra* L. (both known as mulberry) - deciduous trees belonging to the family *Moraceae*. Their various plant parts have been used in traditional Chinese medicine for centuries. The root bark of M. alba and the compounds it possesses anti-allergic, anti-inflammatory, antimicrobial, antioxidant, antiviral, cytotoxic, hypoglycemic, hypolipidemic, and neuroprotective activities. A study by Zelova et al. that isolates root M. alba compounds shows that Sanggenon H reduced the activation of NF-kB transcription factor [9].

TNF alpha is one of the crucial components in the immune response of the human organism. Research by Galo et al. determined serum levels of TNF-alpha in women who underwent laparoscopy or laparotomy due to pelvic pain, infertility, dysmenorrhoea or pelvic tumors due to endometriosis [10]. The TNF-alpha level between the endometriotic and non-endometriotic groups were statistically significant, making it a newfound non-invasive diagnostic marker for endometriosis. In endometriosis, TNF Alpha binds to TRADD (tumor necrosis factor receptor type 1-associated death domain protein) that activates the IKK complex and the NF-kB p50/p65 complex involved in gene transcription that regulates gene transcription for innate immunity, inflammation, and cell survival. This process results in a decrease in the ability of apoptosis in eutopic endometrial cells so that the cells survive [11].

Our study showed that Sanggenon C has the highest affinity to TNF alpha receptor. The binding of Sanggenon C to the TNF-alpha receptor induces apoptosis via the TRAIL (Tumor Necrosis Factor Related Apoptosis Including Ligand) pathway. TRAIL is a part of TNF that selectively induce apoptosis in cancer cells without causing toxicity to normal cells. TRAIL induces apoptosis by interacting with cell death pathways with TRAIL-R1 receptors (death receptor 4-DR4) and TRAIL-R2 (death receptor)-DR5. Sanggenon C is a well-known active benzopyrone agent of the flavonoid derivative with valuable biological properties, including anticancer, anti-inflammatory, antimicrobial, antiviral, antithrombotic, and immune-modulatory activities. A study conducted by Chen et al. showed that Sanggenon C induces apoptosis of colon cancer cells by increasing reactive oxygen species generation and decreasing nitric oxide production, which is associated with inhibition of inducible nitric oxide synthase expression and activation of mitochondrial apoptosis pathway [12].

Meanwhile, in the NF-kB pathway, the Sanggenon H will bind to the p50/p65 complex to activate the apoptotic pathway. Sanggenon H is a prenylated flavonoids which isolated as one of Morus alba (known as mulberry) root bark compounds [7]. Study by Zelova et al. shows that Sanggenon H was assigned as nontoxic compounds based on its IC_50_ values of >10 μM. A review by Wei et al. about bioactive compounds from root barks of Morus plants (Sang-Bai-Pi) refers that Sanggenon H inhibited the secretion of TNF-α, IL-1β and NF-kB nuclear translocation in LPS-stimulated macrophage [13].

Another endometriosis pathogenesis is from the prostaglandin (PGE2) pathway. PGE2 and activation of its receptors has several effects on endometriosis pathogenesis: suppressing the macrophage scavenging capacity, increasing biosynthesis of17-β estradiol (E2) through EP2 receptors, and accelerate cell proliferation through EP3 receptors. When PGE2 attaches to the EP2 receptor, this process activates adenylyl cyclase (AC) to make cyclic AMP (cAMP), which then activates protein kinase A (PKA). Activated PKA translocates to the nucleus and phosphorylates cAMP responsive element-binding protein (CREB), which binds to the StAR gene promoter and aromatase. Increased expression of StAR and aromatase promoter genes induces the production of 17-β estradiol (E2) inside the lesion. By its binding to EP3 receptor PGE2 activates protein kinase Cδ (PKCδ) -Raf-MEK-ERK, which directly increases FGF9 transcription. Overexpression of FGF9 will stimulate endometriosis cell proliferation by autocrine and paracrine regulation [14].

This study showed a successful binding of all active components of Sulawesi Propolis to EP2 dan EP3 receptors, with Epicryptoacetalide has the highest ΔG binding to EP2 receptors and Polydatin to EP3 receptors. Polydatin (PLD), the 3-O-b-glucopyranoside, a well-known stilbenoid compound resveratrol, is a major compound of Fallopia japonica (Houtt.) R. Decr. (Japanese knotweed), which is widely used in traditional Chinese medicine to treat infections, inflammatory diseases and circulatory problems. It is also detected in grapes, peanuts, hop cones, red wines, hop pellets, cocoa-containing products, chocolate products and many daily diets [15]. When administered to endometriosis patients, polydatin will be attached to EP2 receptors to activate the phagocytosis activity which was inhibited by prostaglandin before. It has shown a wide range of biological activities including anti-inflammatory, anti-oxidant, anti-cancer, neuroprotective, hepatoprotective, nephroprotective and immunostimulatory effects. It seems that the mechanisms of the PLD's beneficial effects are related to cellular anti-oxidants and anti-inflammatory cascades. De Maria conducted a study investigating whether the Resveratrol (*trans*-3,5,49-trihydroxystilbene) and its natural precursors Polydatin (resveratrol-3-Ob-mono-D-glucoside, the glycoside form of resveratrol) combination, might have a cooperative antitumor effect on either growing or differentiated human adenocarcinoma colon cancer cells. It showed that the cause of polydatin-induced cell death was apoptosis, as suggested by activation of caspase-3 cystein protease, acting as a common effector pathway for apoptotic processes originating on both cell membrane and mitochondrial levels [16].

Estrogen initiates ectopic endometrial growth and changes in estrogen signalling are associated with endometriosis. The source of estradiol, which promotes the growth of ectopic tissue, is not only obtained from estrogen-producing organs such as the ovaries and adrenals but is also known to be produced locally by the expression of aromatase in endometriosis implant. This ectopic endometrial tissue expresses estrogen receptors (ER) α and β differently than eutopic endometrial tissue, where ERβ is expressed higher. The reduced methylation of the gene promoter that encodes ERβ is thought to produce excessive expression of ERβ in endometriosis, which in turn suppresses ERα expression and reduces the formation of progesterone receptors in endometriosis cells mediated by estradiol. This mechanism contributes to the progesterone resistance of endometriosis cells, seen by the inactivity of genes mediated by the action of progesterone [14].

Progesterone typically triggers an endometrial response characterized by inhibition of epithelial cell proliferation with apoptotic peaks when progesterone levels decrease in the two days before menstruation. Inflammation that occurs in endometriosis can cause progesterone resistance due to competition or interference by pro-inflammatory transcription factors. It is mediated by proteins such as the FKBP4 or Hic-5 companion protein. Progesterone resistance is characterized by a reduced expression of progesterone B receptors compared to progesterone A receptors [17].

Our finding showed that Irilone has the highest affinity towards progesterone receptor B. Irilone is an isoflavonoid found in Red Clover (Trifolium pratense*)*. A study by Lee et al. investigates the use of botanical dietary supplements. They used a progesterone response element (PRE)-luciferase (Luc) reporter assay to identify four phytoprogestins present in a standardized red clover (Trifolium pratense) extract. They found that the component irilone potentiated the effect of progesterone in both endometrial and ovarian cancer cell lines. In these cancers, progesterone action is generally associated with positive outcomes; thus the potentiating effect of irilone may provide entirely new strategies for enhancing progesterone signalling as a means of mitigating conditions such as fibroids and endometriosis [18].

The active compound Epicryptoacetalide of propolis will bind to alpha estrogen receptors. Limited study has been found on Epicryptoacetalide. One study conducted by Hao et al. examined salvia plants, one of the ingredients in traditional Chinese medicine. Epicryptoacetalide is one of the ingredients contained in *Salvia miltiorrhiza* which is known to have properties in removing blood stasis and relieving pain, activating blood to promote menstruation, relieving restlessness; irregular menstruation, amenorrhea, menalgia, mass in the abdomen, stabbing pain in the chest and abdomen, pyretic arthralgia, ulcers and sores, sleeplessness, swelling of the liver and spleen, angina pectoris. But there is no explanation in detail how Epicryptoacetalide works and what effects it causes [19].

The most active compound of Sulawesi Propolis that binds to beta estrogen receptors is Chrysin-7-O-β-d-glucopyranoside, as shown in Table 2. It was found in many plants, such as Calicotome villosa, Halostachys caspica and adenocarpus. An in-silico study conducted by Nganou et al. on adenocarpus plants to colon cancer cells showed that chrysin 7-O-β-d-glucopyranoside had good docking results. This means that chrysin 7-O-β-d-glucopyranoside satisfies all the properties of pharmacological or biological properties with the best result when compared with known standards Capecitabine and 5-Fluorouracil in inhibiting colon cancer cells [20].

Although Sulawesi Propolis showed a strong binding potential towards endometriosis-related receptors, its real biologic effects in endometriosis have not been elucidated by this study. To answer this issue, dynamic molecular docking or in-vitro/vivo studies are needed. An animal study is currently carried out to investigate Propolis’ effect on endometriosis tissue. Only after this then clinical trial in human can be conducted to evaluate the effect of Sulawesi Propolis and its effective dosage in endometriosis.

## 5 Conclusion

Sulawesi Propolis active components have a good binding capacity to several receptors involved in endometriosis pathogenesis. This promising result as the endometriosis drug should be followed by clinical trials to prove its efficacy as an alternative medication for endometriosis.

## Ethical approval

The Ethics Committee of the Faculty of Medicine, University of Indonesia has approved our study protocol in protocol no. 19-10-1269 with date of approval February 24th, 2020.

## Sources of funding

Our study has grant from TADOK 2019 (Tugas Akhir Doktor) from 10.13039/501100006378Universitas Indonesia. The study sponsor had no such involvement in this study.

Herbert Situmorang: Writing the paper.

Andon Hestiantoro: Study concept.

Sigit Purbadi: Study concept.

Darin Flamandita: Data analysis and interpretation.

Muhamad Sahlan: Study concept.

## Registration of research studies

Name of the registry: ClinicalTrials.gov.

Unique Identifying number or registration ID:

ClinicalTrials.gov ID NCT04374006.

Protocol ID 19-10-1269’

Hyperlink to your specific registration (must be publicly accessible and will be checked): https://clinicaltrials.gov/ct2/show/NCT04374006.

## Guarantor

Herbert Situmorang, MD, +62 815-8626-3987, herbert.situmorang@ui.ac.id.

## Consent

We do not have any consent.

## Provenance and peer review

Not commissioned, externally peer reviewed.

## Declaration of competing interest

There is no potential conflicting interest in this study.
